# *In vitro* antibacterial activity of platelet-rich plasma against Gram-positive and Gram-negative pathogens

**DOI:** 10.3389/fcimb.2026.1757780

**Published:** 2026-02-02

**Authors:** Yan Liu, Lei Liu, Jinbing Du, Wenxu Ni, Tingting Zou, Zihan Yuan, Yingkai Xu, Junying Li, Mengyu Shen, Yong Qi, Wanbing Liu

**Affiliations:** 1 Department of Clinical Laboratory, Maternal and Child Health Hospital of Hubei Province, Wuhan, Hubei, China; 2 Department of Transfusion, General Hospital of Central Theater Command, Wuhan, Hubei, China; 3 Medical College, Wuhan University of Science and Technology, Wuhan, Hubei, China; 4 Basic Medical Laboratory, General Hospital of Central Theater Command, Wuhan, Hubei, China

**Keywords:** antibacterial activity, frozen platelet-rich plasma, infected wounds, platelet-poor plasma, platelet-rich plasma

## Abstract

Platelet-rich plasma (PRP) is known as an autologous biological agent that can immensely promote wound healing. Bacterial infection is a common complication of wounds which obstructs wound healing. However, the effect of PRP on bacteria is still inconclusive. This *in vitro* study evaluated the antibacterial activity of PRP, platelet-poor plasma (PPP) and Frozen-PRP (Fro-PRP) against *Escherichia coli*, *Klebsiella pneumoniae*, *Enterobacter cloacae*, *Staphylococcus aureus*, and *Enterococcus faecalis*. The antimicrobial activity was performed using modified agar diffusion method, colony-forming units counting test, and bacterial growth curve analysis. The results showed that PRP, PPP and Fro-PRP had antimicrobial effects on five bacterial strains including *Escherichia coli*, *Klebsiella pneumoniae*, *Enterobacter cloacae*, *Staphylococcus aureus*, and *Enterococcus faecalis*. The antimicrobial activity of PRP was significantly stronger than that of Fro-PRP and PPP. To a certain extent, frozen storage may reduce the antibacterial ability of PRP. Overall, our study demonstrates that PRP and other platelet derived products seem to be a promising alternative tool for infected wounds treatment.

## 1 Introduction

Bacterial infection is a major clinical complication of wounds and is a key contributor to their progression into chronic wounds. Bacterial infection constitutes a major impediment to the wound-healing process. At the injured site, bacteria can multiply and secrete toxins, thereby damaging peri-wound tissues and activating inflammation. If this inflammatory reaction persists, it disrupts all subsequent stages of wound repair. Specifically, in the inflammatory phase, an infection-driven over inflammatory response may extend tissue injury to neighboring healthy areas, aggravate the original lesion, and result in tissue necrosis, fibrosis, and suboptimal angiogenesis. During the proliferative phase of wound healing, bacterial infection inhibits the functions of fibroblasts, keratinocytes, and endothelial cells, all of which are essential for effective tissue repair. During the subsequent remodeling phase, bacterial infection further disrupts the balance between collagen synthesis and degradation. Eventually, this renders the wound difficult to heal and promotes its progression to a chronic wound. Numerous studies have demonstrated that the presence of bacterial infection prolongs the wound-healing period, thereby increasing patients’ pain and healthcare costs. Moreover, chronic wounds not only adversely affect patients’ quality of life but also predispose them to severe systemic infections, such as sepsis, which can be life-threatening (Edwards and Harding, 2004; Percival et al., 2012; Scali and Kunimoto, 2013).

*Escherichia coli* (*E. coli*), *Klebsiella pneumoniae* (*K. pneumoniae*), *Enterobacter cloacae* (*E. cloacae*), *Staphylococcus aureus* (*S. aureus*), and *Enterococcus faecalis* (*E. faecalis*) are among the most frequently identified pathogens in wound infections. Patients who are elderly, immunocompromised, diabetic, obese, cognitively impaired, or have sustained trauma are at increased risk of developing such infections. Furthermore, the overuse of antibiotics has promoted the emergence of multidrug-resistant organisms, which can cause life-threatening opportunistic infections. Therefore, developing novel antibacterial strategies and optimizing existing therapeutic approaches are essential for more effective management of infected wounds.

Platelet-rich plasma (PRP) may offer a novel adjunctive approach for the management of wound infections. PRP is an autologous preparation obtained from the patient’s whole blood by centrifugation. It is a composite biological product that contains multiple bioactive components including platelets, leukocytes, fibrin, etc. PRP can release a broad spectrum of biologically active substances such as growth factors, cytokines and antimicrobial proteins, which collectively enhance tissue repair, attenuate inflammation, and modulate immune function (Oudelaar et al., 2019). In recent years, PRP has been widely used in clinical fields such as orthopedics, dentistry, plastic surgery, and reproductive medicine because of its regenerative and anti-inflammatory properties (Foster et al., 2009). Serraino et al (Serraino et al., 2015). and Patel et al (Patel et al., 2016). pointed out that PRP not only reduced the incidence of deep wound infection and sternotomy-related complications but also facilitated wound healing and lowered the medical costs associated with preventing wound infection. Therefore, in addition to its wound-healing potential, the putative antibacterial effect of PRP has become a current focus of research. Although several recent studies have indicated that PRP can influence bacterial growth (Sethi et al., 2021), its antimicrobial spectrum remains unclear. In addition, its antibacterial activity has not been fully elucidated, and the findings reported to date are inconsistent. For example, Burnouf et al. found that PRP inhibited the growth of *S. aureus*, *E. coli*, *K. pneumoniae*, and *Pseudomonas aeruginosa* (*P. aeruginosa*), but showed no inhibitory effect on *E. cloacae*, *Bacillus cereus*, *Bacillus subtilis*, and *Staphylococcus epidermidis* (Burnouf et al., 2013). In contrast, Li et al. established an infected animal model and used a bacterial kill-curve assay to analyze the antimicrobial activity of PRP against several bacterial strains both *in vivo* and *in vitro*. They concluded that PRP markedly inhibited *S. aureus, Group A streptococcus* and *Neisseria gonorrhoea*, but exerted no antimicrobial effect against *E. coli* and *P. aeruginosa* (Li et al., 2013). Taken together, these findings indicated that the antibacterial spectrum of PRP remains uncertain, as different studies have yielded discrepant results. Therefore, further investigations are warranted. In this study, we aimed to clarify the *in vitro* antimicrobial effects of PRP against Gram-negative bacteria including *E. coli*, *K. pneumoniae*, and *E. cloacae*, as well as Gram-positive bacteria including *S. aureus* and *E. faecalis*. A further objective was to compare the antibacterial activities of PRP with platelet-poor plasma (PPP) and Frozen-PRP (Fro-PRP).

## 2 Materials and methods

### 2.1 Bacterial strain

*Escherichia coli* (*E. coli*, ATCC 25922)*, Klebsiella pneumoniae* (*K. pneumoniae*, ATCC 700603)*, Enterobacter cloacae* (*E. cloacae*, ATCC 700323), *Staphylococcus aureus* (*S. aureus*, ATCC 29213), and *Enterococcus faecalis* (*E. faecalis*, ATCC 29212) were obtained from the Microbiology Laboratory of the Department of Laboratory Medicine at the General Hospital of the Central Theater Command. *S. aureus* was cultured in tryptic soy broth (TSB), whereas the other strains were cultured in brain heart infusion (BHI) medium. All strains were incubated in a shaking incubator at 220 r/min and 37 °C.

### 2.2 Preparation of PRP, PPP and Fro-PRP

Fifty-seven volunteers were recruited for this study, and written informed consent was obtained from each participant. The study was approved by the Ethics Committee of the General Hospital of the Central Theater Command. Individuals were excluded if they presented with infection, had used non-steroidal anti-inflammatory drugs within 5 days prior to blood donation, had serious cardiovascular diseases, or other diseases that made them unsuitable for blood cell separation. Before blood collection, a routine blood test was performed. Donors with hemoglobin values < 120 g/L or platelet values < 120×10^9^/L were excluded.

PRP was prepared using an automated apheresis system of (Trima Accel, Terumo BCT, USA) according to our previously established protocol (Liu et al., 2024). All resultant preparations were classified as leukocyte-poor PRP. Each sample was subsequently divided into three equal aliquots, designated as fresh PRP, frozen PRP, and PPP. Fresh PRP was maintained at 22 °C, while frozen PRP was stored at -80 °C and subsequently rewarmed at 37 °C before use. PPP was produced by subjecting PRP to high-speed centrifugation and collecting the plasma supernatant. PRP, PPP and Fro-PRP were used without additional activation in this study.

### 2.3 Modified agar diffusion method

A modified agar diffusion method was used to evaluate bacterial growth inhibition. Bacterial suspension of the five tested strains were adjusted to 0.5 McFarland (1×10^8^ CFU/mL) and evenly spread into Mueller-Hinton agar (MH agar) plates. Four wells were punched in each plate. Subsequently, 50 μL of PRP, PPP, Fro-PRP, and 0.9% NaCl was added to the respective wells. The plates were allowed to stand for 10–15 min to permit diffusion and then incubated at 37 °C for 6–10 h. The diameters of the inhibition zones were then measured. By increasing the PRP loading volume and shortening the observation period in this modified agar diffusion method, we were able to obtain clearer experimental observations and better photographic imaging quality.

### 2.4 Colony-forming unit counting assay

The bacterial suspensions of the five test strains were adjusted to 0.5 McFarland (1×10^8^ CFU/mL). Subsequently, these suspensions were co-cultured with PRP, PPP, Fro-PRP, or BHI/TSB (blank control) according to the reaction system described in **Table 1**. At 0 h, 2 h, 4 h, and 6 h, 20 μL of the co-culture mixture was collected and subjected to ten-fold serial dilution. 10 μL of each dilution was then spotted onto Luria-Bertani (LB) agar plates in triplicate. The plates were incubated inverted at 37 °C for 24 h, after which the CFUs on each plate were counted.

**Table 1:** The reaction system of the colony-forming units counting test.

Composition Group	Bacterial suspensions	PRP	Fro-PRP	PPP	BHI/TSB	Total
Control group	200 μL	–	–	–	1.8 mL	2.0 mL
PRP group	200 μL	500 μL	–	–	1.3 mL	2.0 mL
Fro-PRP group	200 μL	–	500 μL	–	1.3 mL	2.0 mL
PPP group	200 μL	–	–	500 μL	1.3 mL	2.0 mL

-:Means there was no such component.

### 2.5 Bacterial growth curve assay

Five bacterial strains were cultured in BHI or TSB medium at 37 °C until the optical density at 600 nm (OD_600_) reached approximately 0.8. According to the reaction system presented in **Table 2**, the bacterial cultures were diluted 1:100 in fresh medium containing PRP, PPP, and Fro-PRP and then incubated in 96-well plates at 37 °C. Bacterial cultures diluted 1:100 in fresh BHI or TSB without any additive served as controls. Bacterial growth was monitored by measuring OD_600_ values at predefined time points. All experiments were performed on three independent cultures, and a representative result was presented.

**Table 2:** The reaction system of the bacterial growth curve method.

Composition Group	Bacterial suspensions	PRP	Fro-PRP	PPP	BHI/TSB
Control group	2 μL	–	–	–	200 μL
PRP group	2 μL	50 μL	–	–	150 μL
Fro-PRP group	2 μL	–	50 μL	–	150 μL
PPP group	2 μL	–	–	50 μL	150 μL

-:Means there was no such component.

### 2.6 Statistical analysis

Sample size was calculated by *a priori* power analysis (G*Power, version 3.1.9.78). Based on our preliminary data and relevant published literature (Çetinkaya et al., 2019; Gilbertie et al., 2020), a minimum of 9 volunteers was needed to achieve a statistical power of 0.80 at a significance level of 0.05. In this study, all experiments were conducted 19 biological replicates (independent donors), with 3 technical replicates per sample. Statistical data were presented as mean ± standard deviation (SD). Independent samples t-tests were used for pairwise comparisons. For comparisons among multiple groups, one-way analysis of variance (ANOVA) was performed, followed by least significant difference (LSD) or Tamhane’s *post hoc* tests, as appropriate. Statistical analyses were conducted using SPSS 26.0 software, and graphs were generated with GraphPad Prism 9 software. A two-sided *P* < 0.05 was considered statistically significant.

## 3 Results

### 3.1 Characterization of PRP

The concentrations of platelets, white blood cells (WBCs) and red blood cells (RBCs) in whole blood were (236.50 ± 48.13) ×10^9^/L, (6.44 ± 1.50) ×10^9^/L, and (4.94 ± 0.72) ×10^12^/L, respectively. The platelet concentration in PRP was (1288.15 ± 470.99) ×10^9^/L, representing a 5.5-fold increase compared with whole blood from the same donors. The blood cells concentration in Fro-PRP was comparable to that of PRP. In contrast, the platelets concentration in PPP was (13.85 ± 18.91) ×10^9^/L, which was 17.1-fold lower than that of whole blood, and the difference was statistically significant. Moreover, in both PRP and PPP, WBCs and RBCs counts were significantly lower than those in whole blood (**Table 3**).

**Table 3:** Comparison of platelet, white blood cell and red blood cell counts in whole blood, PRP and PPP.

Group	PLT (×10^9/^L)	WBC (×10^9/^L)	RBC(×10^12/^L)
Whole blood	236.50 ± 48.13	6.44 ± 1.50	4.94 ± 0.72
PRP	1288.15 ± 470.99*	0.12 ± 0.28*	0.04 ± 0.04*
PPP	13.85 ± 18.91^#&^	0.00 ± 0.01^#^	0.00 ± 0.01^#&^

*: There was a significant difference between Whole blood and PRP (*P*< 0.001). #: There was a significant difference between Whole blood and PPP (*P*< 0.001). &: There was a significant difference between PRP and PPP (*P*< 0.001).

### 3.2 Inhibition zones analysis

**Figure 1:**
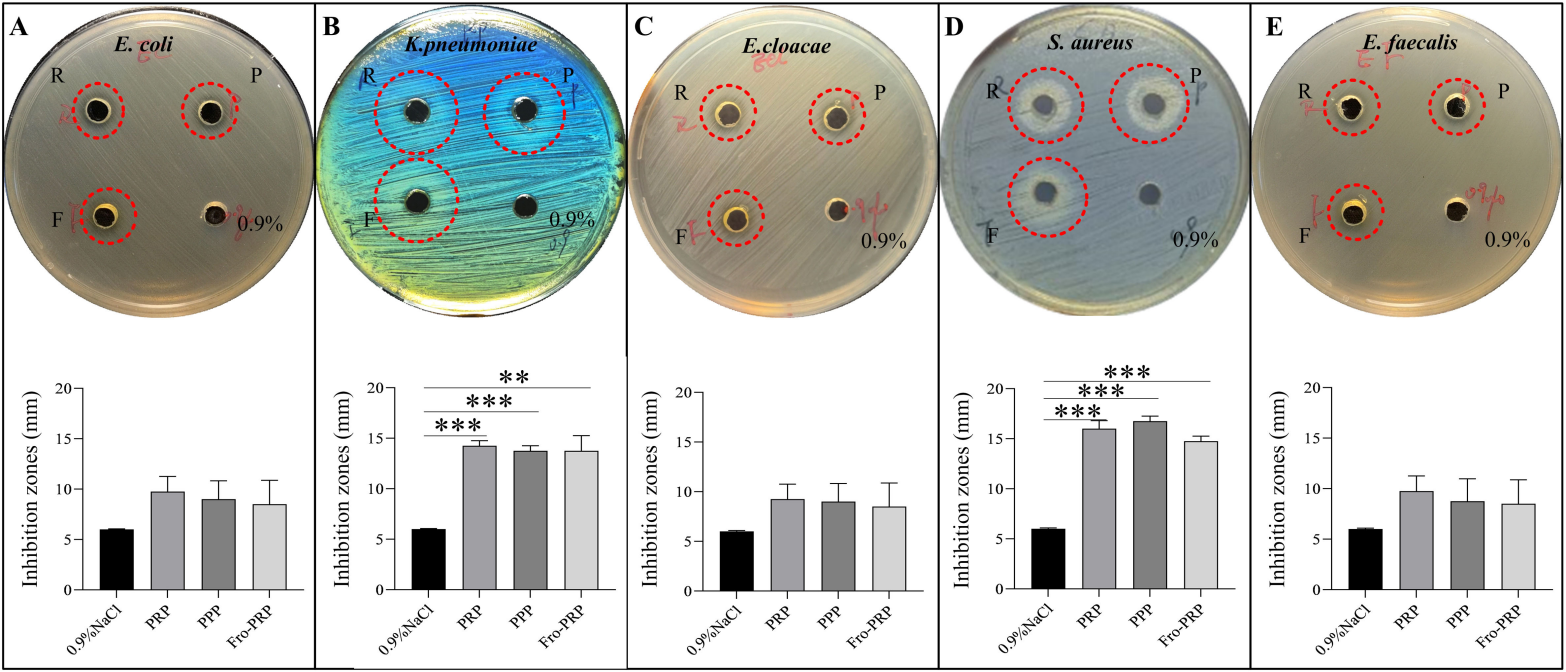
Photos and quantification of the inhibition zones of PRP, PPP and Fro-PRP incubated with *E*. *coli***(A)**, *K*. *pneumoniae***(B)**, *E*. *cloacae***(C)**, *S. aureus***(D)**, and *E. faecalis***(E)**. R, Platelet-rich plasma; P, platelet-poor plasma; F, Frozen platelet-rich plasma; 0.9%, 0.9%NaCl; **P<0.01, ***P<0.001.

The *in vitro* antibacterial effects of PRP, PPP, and Fro-PRP against the five bacterial strains, assessed using the modified agar diffusion method, were shown in **Figure 1**. Compared with the 0.9% NaCl control group, all three preparations (PRP, PPP, and Fro-PRP) produced visible inhibition zones on agar plates for each of the five bacterial strains. Notably, the median diameters of the inhibition zones against *K. pneumoniae* and *S. aureus* in the PRP, PPP, and Fro-PRP groups were significantly larger than those in the 0.9% NaCl group (**Figures 1B, D**). However, for *E. coli*, *E. cloacae*, and *E. faecalis*, no statistically significant differences in inhibition zone diameters were observed between PRP-, PPP-, or Fro-PRP-treated plates and the 0.9% NaCl control (**Figures 1A, C, E**).

### 3.3 Colony-forming unit counts

Bacterial strains were co-cultured with PRP, PPP, and Fro-PRP, and bacterial proliferation was evaluated by comparing CFU counts at 2, 4, and 6 hours with the baseline CFU counts at 0 hour.

**Figure 2:**
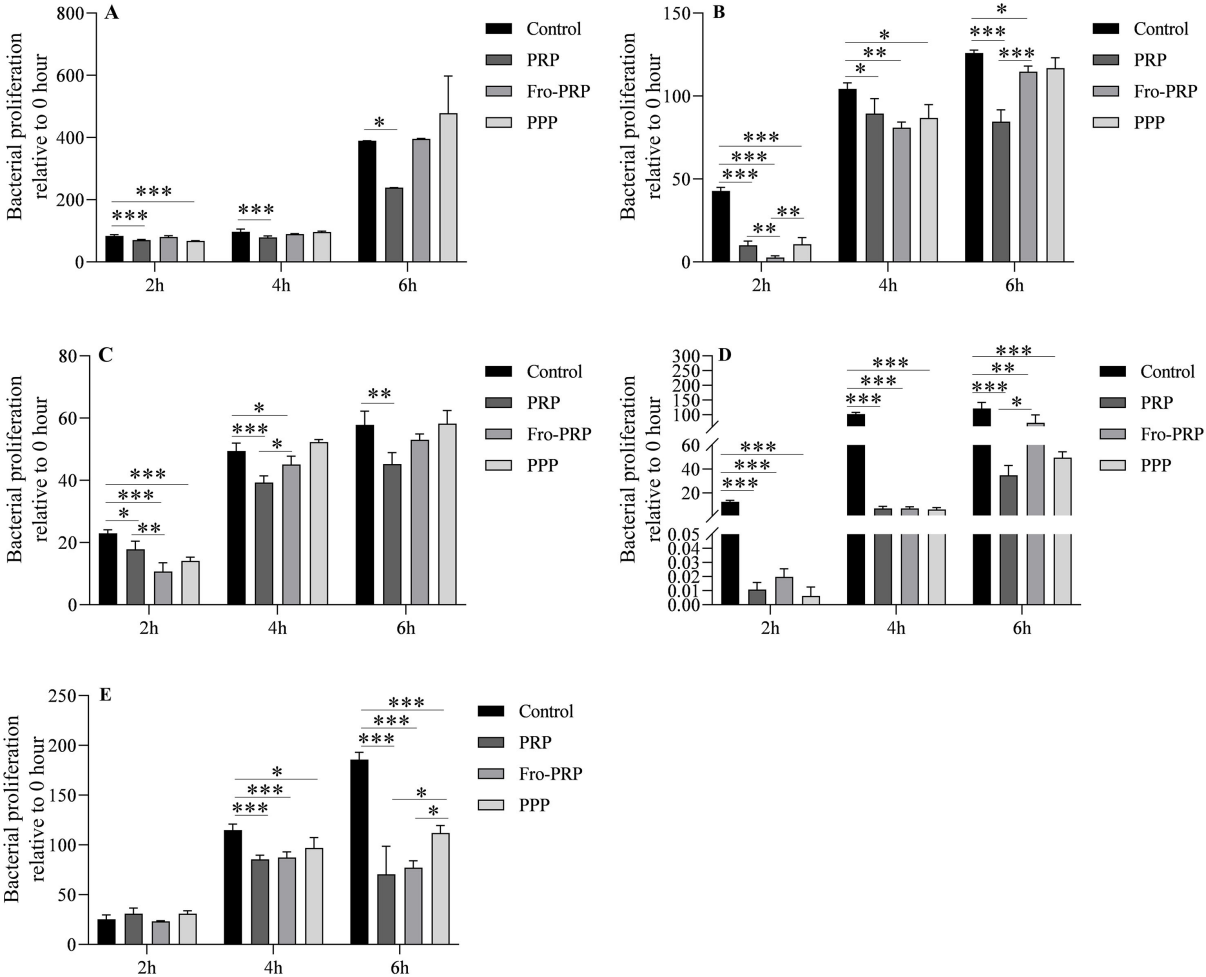
Antibacterial properties of PRP, PPP and Fro-PRP against *E. coli***(A)**, *K*. *pneumoniae***(B)**, *E*. *cloacae***(C)**, *S. aureus***(D)**, and *E. faecalis***(E)**, measured by colony-forming units counting test. *P<0.05, **P<0.01, ***P<0.001.

Compared with the control group, PRP significantly inhibited the proliferation of *E. coli* at 2, 4, and 6 hours. PPP significantly inhibited the proliferation of *E. coli* at 2 hours, but no significant differences were observed at 4 or 6 hours. Fro-PRP showed no detectable antimicrobial effect against *E. coli* (**Figure 2A**).

Compared with the control group, PRP significantly inhibited the growth of *K. pneumoniae* at 2, 4, and 6 hours. PPP also significantly suppressed *K. pneumoniae* growth at 2 and 4 hours, but this effect was no longer evident at 6 hours. Fro-PRP significantly inhibited the proliferation of *K. pneumoniae* at 2, 4, and 6 hours. In addition, at 2 hours, the antibacterial effect of Fro-PRP was significantly better than that of PRP and PPP, whereas at 6 hours, PRP exhibited a stronger antibacterial effect than Fro-PRP (**Figure 2B**).

Compared with the control group, PRP significantly inhibited the growth of *E. cloacae* at 2, 4, and 6 hours. PPP significantly inhibited the proliferation of *E. cloacae* only at 2 hours, with no significant differences at 4 or 6 hours. Fro-PRP significantly inhibited *E. cloacae* at 2 and 4 hours, but this effect was not observed at 6 hours. In addition, at 2 hours, Fro-PRP showed a significantly stronger antibacterial effect than PRP, whereas at 4 hours, PRP exhibited greater antibacterial activity than Fro-PRP (**Figure 2C**).

*S. aureus* was significantly inhibited by PRP, PPP, and Fro-PRP at 2, 4, and 6 hours compared with the control. In addition, at 6 hours, PRP exhibited a significantly stronger antibacterial effect than Fro-PRP (**Figure 2D**).

*E. faecalis* was significantly inhibited by PRP, PPP, and Fro-PRP at 4,and 6 hours, whereas no statistical difference was observed at 2 hours. Moreover, the antibacterial effects of PRP and Fro-PRP were significantly greater than that of PPP at 6 hours (**Figure 2E**).

### 3.4 Bacterial growth curve

**Figure 3:**
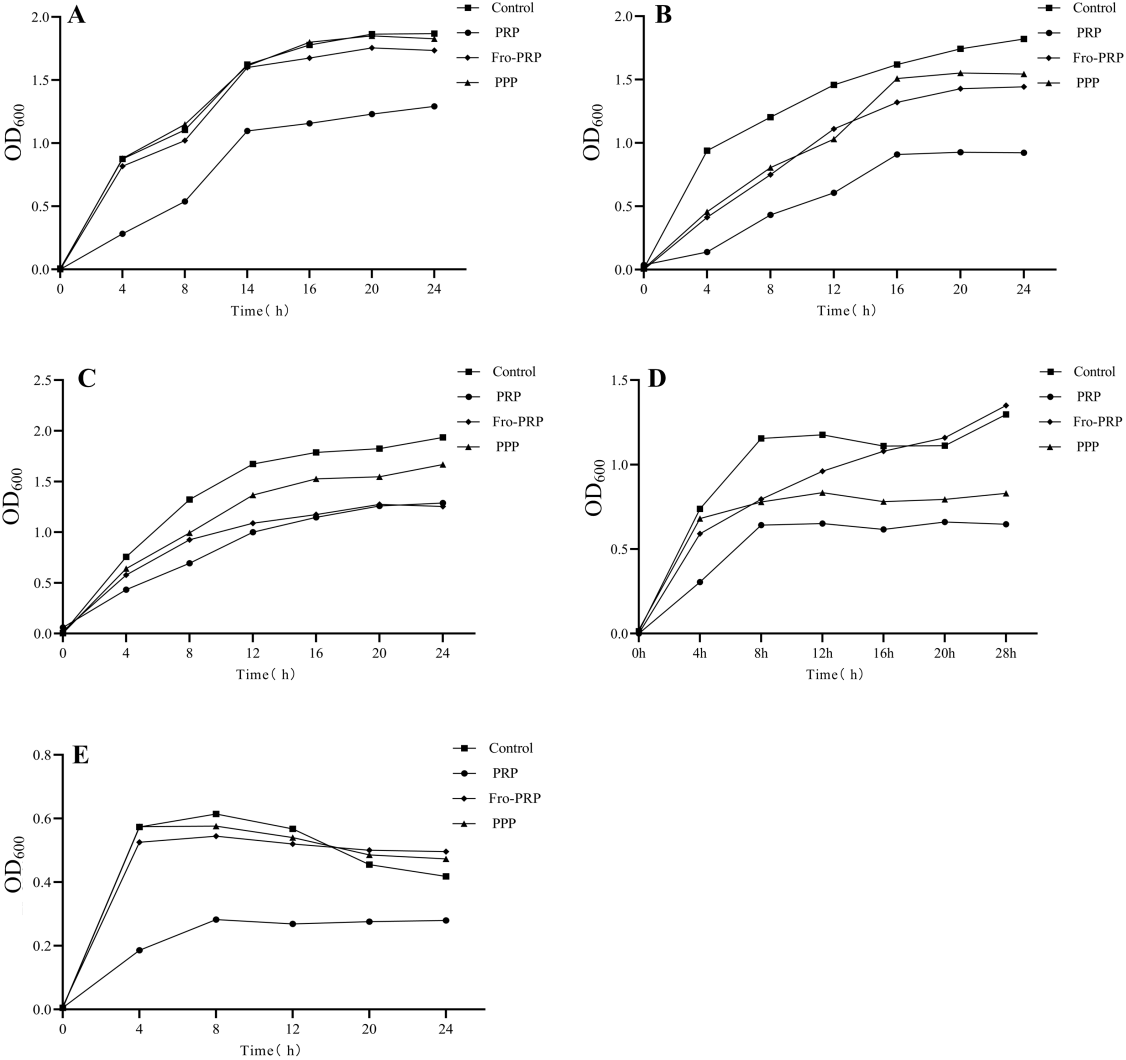
Antibacterial properties of PRP, PPP and Fro-PRP against *E.coli***(A)**, *K.pneumoniae***(B)**, E.cloacae **(C)**, S.aureus **(D)**, and *E. faecalis***(E)**, measured by bacterial growth curve method test.

According to the growth curves, PRP significantly inhibited the growth of all five bacterial strains (*E. coli*, *K. pneumoniae*, *E. cloacae*, *S. aureus*, and *E. faecalis*) compared with the control (**Figure 3**). Moreover, the inhibitory effect of PRP was more pronounced than that of PPP and Fro-PRP. Fro-PRP significantly inhibited *E. coli*, but its effect was weaker than that of PRP, whereas PPP did not exhibit a significant inhibitory effect on *E. coli*. Both Fro-PRP and PPP significantly inhibited the growth of *K. pneumoniae* and *E. cloacae.* For *S. aureus*, PPP produced a significant inhibitory effect throughout the observation period, while Fro-PRP exhibited a significant inhibitory effect on its growth only before 16 hours and showed no inhibition thereafter. For *E. faecalis*, Fro-PRP inhibited its growth within the first 12 hours, but showed no antibacterial effect after 12 hours. PPP inhibited the growth of *E. faecalis* within 8 to 12 hours but had no antibacterial effect beyond 12 hours.

## 4 Discussion

Bacterial infection is one of the most common complications of wounds and represent a major barrier to wound healing. Infected wounds arise when invading bacteria disrupt the cutaneous barrier and induce cellular necrosis or apoptosis. The pathogens also trigger an excessive inflammatory response within the wound bed, which further delays the healing process (Malone and Schultz, 2022; Swanson et al., 2022). Consequently, the development of strategies that can simultaneously promote chronic wound repair and prevent bacterial infection has become a major focus of current research in the medical field. PRP has been extensively investigated over the past decades for its regenerative properties. On the contrary, relatively few studies have examined the antimicrobial effects of PRP and its derivatives, and the available findings remain inconsistent. Bieleck et al. showed that platelet-rich plasma gel (PRG), a derivative of PRP, exhibited strong *in vitro* antibacterial activity against *S. aureus* and *E. coli*, but had no inhibitory effect on *E. faecalis*, *P. aeruginosa*, and *K. pneumoniae*. Moreover, PRG even appeared to promote the growth of *P. aeruginosa*, which could potentially exacerbate inflammation (Bielecki et al., 2007). Li et al. found that PRG exhibited significant *in vitro* antibacterial activity against methicillin-susceptible *Staphylococcus aureus* (MSSA), methicillin-resistant *Staphylococcus aureus* (MRSA), *Neisseria gonorrhoeae*, and group A *streptococcus*, but had no inhibitory effect on *E. coli* and *P. aeruginosa* (Li et al., 2013). Chen et al. further showed that PRG inhibited the growth of *S. aureus* but did not suppress *P. aeruginosa* and *E. coli* (Chen et al., 2013). Çetinkaya et al. found that PRP exerted inhibitory activity against MRSA, carbapenem resistant *P. aeruginosa*, and broad-spectrum β-lactase producing *K. pneumoniae* but not against vancomycin resistant *Enterococcus* (VRE) (Çetinkaya et al., 2019). Drago et al. reported that PRP inhibited the growth of *E. faecalis*, *Candida albicans*, *Streptococcus agalactiae* and *Streptococcus oralis*, but not against *P. aeruginosa* (Drago et al., 2013).

We speculated that the heterogeneous and sometimes contradictory antibacterial profiles of PRP observed across studies may arise from methodological differences, including the PRP preparation technique, the final platelet concentration in PRP, and the specific antimicrobial testing procedures employed. Melo et al. concluded that platelets concentration and ratio constituted the essential basis for subsequent PRP research (De Melo et al., 2018). In the present study, the platelet count in PRP was 5.5 times higher than that in whole blood, thereby establishing the conditions required for reliable *in vitro* antimicrobial assays. We assessed the antibacterial effect of PRP against *E. coli*, *K. pneumoniae*, *E. cloacae*, *S. aureus* and *E. faecalis* using three *in vitro* antibacterial methods including a modified agar diffusion method, colony-forming units counting test, and bacterial growth curve analysis.

In our study, the modified agar diffusion assay showed that PRP produced inhibition zones with significant antibacterial activity against *K. pneumoniae* and *S. aureus*. PRP also formed small inhibitory rings against *E. coli*, *E. cloacae* and *E. faecalis*, but these effects were not statistically different from those observed with 0.9% NaCl. In the same assay, the antibacterial activities of PPP and Fro-PRP were comparable to that of PRP. Çetinkaya et al. reported that PRP showed antibacterial activity against *P. aeruginosa*, *K. pneumoniae*, and VRE using the Kirby-Bauer disc-diffusion method, but showed no antibacterial activity against MRSA (Çetinkaya et al., 2019). In contrast, Cieślik et al. found that L-PRP inhibited the growth of *S. aureus* (both MRSA and MSSA)*, E. faecalis*, and *P. aeruginosa*, but had no inhibitory activity against *E. coli* or *K. pneumoniae* (Cieślik-Bielecka et al., 2018).Aboelsaad et al. declared that only activated PRP effectively inhibited microbial growth (Aboelsaad et al., 2025) Overall, our findings were not entirely consistent with those of these studies, which may be attributable to differences in PRP preparation methods, platelet counts in PRP, the volume of PRP applied, and whether the PRP contained leukocytes or was activated. In our study, we used an modified agar diffusion method with 50 μL of non-activated PRP, which was prepared by an apheresis technique as described in our previous research (Liu et al., 2024). The PRP was leukocyte-poor and had an average platelet count of (1288.15 ± 470.99) ×10^9^/L.

In the CFU counting assay and bacterial growth curve analysis, the bacterial strains were co-cultured with PRP, PPP, or Fro-PRP. We assessed the antimicrobial effects of these three preparations on the five bacteria species at multiple time points. In the CFU counting assay, for *E. coli*, PRP inhibited its growth from 2 h to 6 h, PPP showed an inhibitory effect at 2 h only, and Fro-PRP showed no detectable antibacterial activity. For *K. pneumoniae*, both PRP and Fro-PRP inhibited bacterial growth from 2 h to 6 h, whereas PPP exerted inhibition at 2 h and 4 h. Notably, the antibacterial effect of Fro-PRP at 2 h was significantly superior to that of PRP and PPP, whereas at 6 h the antibacterial effect of PRP was significantly greater than that of Fro-PRP. For *E. cloacae*, PRP inhibited its growth from 2 h to 6 h, Fro-PRP inhibited its growth at 2 h and 4 h, and PPP showed inhibition at 2 h only. Furthermore, the antibacterial effect of Fro-PRP at 2 h was significantly greater than that of PRP, whereas at 4 h PRP exhibited a significantly stronger effect than Fro-PRP. For *S. aureus*, PRP, PPP, and Fro-PRP all inhibited bacterial growth from 2 h to 6 h, and the antibacterial effect of PRP at 6 h was significantly greater than that of Fro-PRP. For *E. faecalis*, PRP, PPP, and Fro-PRP all showed antibacterial activity at 4 h and 6 h, but none of them exhibited an inhibitory effect at 2 h. In addition, the antibacterial effects of PRP and Fro-PRP at 6 h were significantly greater than that of PPP. The lack of detectable antibacterial activity of PRP at 2 h was likely attributable to the slow proliferation of *E. faecalis* in both the control group and experimental groups. Overall, analysis of all assays indicated that PRP displayed stronger antibacterial activity than PPP and Fro-PRP and that its effect was longer lasting. Fro-PRP showed a relatively potent antibacterial effect within the initial 2 h, but the duration of inhibition was shorter than that observed for PRP. In the bacterial growth curve experiment, our study demonstrated that PRP had sustained antibacterial activity against *E. coli*, *K. pneumoniae*, *E. cloacae*, *S. aureus*, and *E. faecalis* from 0 h to 24 h, and for some strains up to 28 h. This observation is consistent with the findings of Çetinkaya et al, who reported that the antimicrobial effect of PRP could persist for up to 10 h (Çetinkaya et al., 2019). Moreover, we found that the antibacterial effect of PRP was significantly greater than that of PPP and Fro-PRP. Notably, PPP showed no antibacterial effect on *E. coli*, whereas Fro-PRP exhibited only weak antibacterial effect against this organism. In the case of *S. aureus*, Fro-PRP no longer exhibited antibacterial effect after 12 h. For *E. faecalis*, both PPP and Fro-PRP showed weak antibacterial effects and even slightly promoted bacterial growth after 12 h. Furthermore, it is noteworthy that regardless of whether PRP, PPP or Fro-PRP was used the OD values of bacteria continued to increase over time, indicating that PRP-derived products exerted predominantly bacteriostatic rather than bactericidal effects. This observation is consistent with the conclusions of the previous review (Varshney et al., 2019).

The mechanism underlying the antibacterial effect of PRP have not yet been fully elucidated. In terms of its composition, platelets represent the predominant and biologically relevant component of PRP. Beyond their central role in thrombosis and hemostasis, platelets also participate in inflammation and the host response to infection. Platelets contribute to antimicrobial defense through two main mechanisms: first, by directly engaging with pathogens and immune cells; and second, by secreting antimicrobial mediators such as immunomodulatory cytokines, chemokines, and antimicrobial peptides, which regulate immune cell functions and support the host immune response (Jenne et al., 2013; Deppermann and Kubes, 2018; Smith, 2022). Aktan et al. evaluated the antimicrobial effects of equine PRP against *S. aureus* and *E. coli*, and demonstrated that equine platelets were capable of releasing reactive oxygen species (ROS), which could contribute to bacterial killing (Aktan et al., 2013). Etulain et al. and Fabbro et al. indicated that platelets in PRP can release some antibacterial peptides including kinins (PF4 (CXCL4)), platelet basic protein (CXCL7), defensins (human β-defensin 2), thymosin β 4 (Tβ4), antimicrobial peptides (fibrinopeptide A or fibrinopeptide B), and interleukin 8 (CXCL-8) (Tohidnezhad et al., 2012; Fabbro et al., 2016; Etulain, 2018). In addition, plasma is another major constituent of PRP. It provides complement factors that, upon activation, mediate bacterial lysis and facilitate the recruitment of leukocytes via the complement cascade (Chen et al., 2017). The contribution of leukocytes to the antibacterial activity of PRP remains controversial. Several authors have proposed that the presence of leucocytes enhances its antimicrobial potential. However, other authors have found that adding leukocytes to PRP does not improve the antimicrobial properties (Drago et al., 2013). In addition, an author has argued that leukocytes may not be able to perform their normal functions when they are taken out of the bloodstream to prepare platelet concentrates and are subsequently placed directly onto the surgical or wound area, since this application bypasses the physiological migratory phase (Varshney et al., 2019). In our study, all PRP preparations were of the pure platelet-rich plasma (P-PRP), also known as leukocyte-poor PRP. Therefore, leukocytes were not included within the scope of our analysis in this study, and the observed antibacterial effects were considered to be mainly attributable to the platelet and plasma components.

The results of the modified agar diffusion assay were not fully consistent with those obtained from the CFU counting test and the bacterial growth curve method. We speculated that in the modified agar diffusion experiment, only the antimicrobial factors released from the plasma fraction of PRP, PPP, and Fro-PRP were able to diffuse into the agar, whereas the platelets present in PRP and Fro-PRP themselves could not migrate through the agar matrix. This is consistent with other studies, suggesting that the bacteriostatic antimicrobial effect of platelet-derived preparations may be attributable mainly to plasma components, such as complement, rather than to the platelets themselves (Drago et al., 2014; Perego et al., 2024). We speculated that, in the CFU counting tests and bacterial growth curve method, PRP derivatives were able to diffuse more freely in the liquid medium, allowing their antimicrobial factors to bind more readily to bacteria and exert effective inhibitory activity. Under these conditions, both the platelet-derived components and the plasma fraction appeared to contribute substantially to the antibacterial effect. Nevertheless, we could not fully account for the inconsistent findings obtained from the CFU test and the bacterial growth-curve analysis, despite the fact that both assays used the same co-culture system of bacteria and PRP derivatives. A plausible explanation is that the two methods differ in their sensitivity to detect antibacterial effects.

In summary, our results revealed that PRP, PPP, and Fro-PRP all had antimicrobial effects against the five tested bacterial strains such as *E. coli*, *K. pneumoniae*, *E. cloacae, S. aureus*, and *E. faecalis*. Based on the *in vitro* experiments performed in this study, the antimicrobial activity of PRP was significantly stronger than that of Fro-PRP and PPP. The inconsistent antimicrobial effects of PRP described in the present and previous studies are most probably related to methodological variability. Variations in PRP preparation protocols, platelet and leukocyte content, activation status, as well as the choice of bacterial species and the initial bacterial inoculum may all contribute to the conflicting findings observed across studies. However, most *in vitro* antibacterial studies, including ours, support the view that PRP exerts antibacterial activity against common wound pathogens, such as *E. coli*, *K. pneumoniae*, *E. cloacae, S. aureus*, and *E. faecalis*. Meanwhile, PRP and its derivatives should be regarded as agents that possess both regenerative and antibacterial properties. The use of PRP in infected wounds should not be regarded as a contraindication. However, before PRP can be widely applied in clinical practice, its antibacterial spectrum needs to be more comprehensively defined, and its underlying antimicrobial mechanisms should be further elucidated.

## 5 Conclusion

PRP, PPP, and Fro-PRP exhibited distinct *in vitro* antimicrobial effects against representative Gram-positive (*S. aureus* and *E. faecalis*) and Gram-negative bacteria (*K. pneumoniae*, *E. coli* and *E. cloacae*). Among these preparations, PRP showed markedly greater antimicrobial potency than Fro-PRP and PPP. To a certain extent, frozen storage may reduce the antibacterial ability of PRP, especially its long-term antibacterial ability. In addition, PRP did not act equally against all tested microorganisms; its inhibitory effects were more pronounced against *K. pneumoniae* and *S. aureus*. However, these *in vitro* findings need to be substantiated by *in vivo* antimicrobial studies and rigorously designed randomized clinical trials. Overall, our study indicates that PRP and PRP-derived preparations could serve as promising supplementary therapies for the treatment of infected wounds.

## Data Availability

The original contributions presented in the study are included in the article/supplementary material. Further inquiries can be directed to the corresponding authors.

## References

[B1] Aboelsaad E. Moustafa S. Amine A. Deghady A. El-Attar L. (2025). Platelet-rich plasma as a potential antimicrobial agent against multidrug-resistant bacteria in diabetic foot infections. Sci. Rep. 15, 15145. doi: 10.1038/s41598-025-97418-0, PMID: 40307308 PMC12043966

[B2] Aktan Í. Dunkel B. Cunningham F. M. (2013). Equine platelets inhibit E. coli growth and can be activated by bacterial lipopolysaccharide and lipoteichoic acid although superoxide anion production does not occur and platelet activation is not associated with enhanced production by neutrophils. Veterinary Immunol. Immunopathol. 152, 209–217. doi: 10.1016/j.vetimm.2012.12.007, PMID: 23332730

[B3] Bielecki T. M. Gazdzik T. S. Arendt J. Szczepanski T. Kròl W. Wielkoszynski T. (2007). Antibacterial effect of autologous platelet gel enriched with growth factors and other active substances: AN *IN VITRO* STUDY. J. Bone Joint Surg. Br. 89-B, 417–420. doi: 10.1302/0301-620X.89B3.18491, PMID: 17356164

[B4] Burnouf T. Chou M. Wu Y. Su C. Lee L. (2013). Antimicrobial activity of platelet (PLT)-poor plasma, PLT-rich plasma, PLT gel, and solvent/detergent-treated PLT lysate biomaterials against wound bacteria. Transfusion 53, 138–146. doi: 10.1111/j.1537-2995.2012.03668.x, PMID: 22563709

[B5] Çetinkaya R. A. Yenilmez E. Petrone P. Yılmaz S. Bektöre B. Şimsek B. . (2019). Platelet-rich plasma as an additional therapeutic option for infected wounds with multi-drug resistant bacteria: *in vitro* antibacterial activity study. Eur. J. Trauma Emerg. Surg. 45, 555–565. doi: 10.1007/s00068-018-0957-0, PMID: 29700554

[B6] Chen J. Losos M. Yang S. Li J. Wu H. Cataland S. (2017). Increased complement activation during platelet storage. Transfusion 57, 2182–2188. doi: 10.1111/trf.14215, PMID: 28671303

[B7] Chen L. Wang C. Liu H. Liu G. Ran X. (2013). Antibacterial effect of autologous platelet-rich gel derived from subjects with diabetic dermal ulcers. In Vitro J. Diabetes Res. 2013, 1–5. doi: 10.1155/2013/269527, PMID: 23671863 PMC3647554

[B8] Cieślik-Bielecka A. Bold T. Ziółkowski G. Pierchała M. Królikowska A. Reichert P. (2018). Antibacterial activity of leukocyte- and platelet-rich plasma: an *in vitro* study. BioMed. Res. Int. 2018, 1–8. doi: 10.1155/2018/9471723, PMID: 30050949 PMC6040244

[B9] De Melo B. A. G. Martins Shimojo A. A. Marcelino Perez A. G. Duarte Lana J. F. S. Andrade Santana M. H. (2018). Distribution, recovery and concentration of platelets and leukocytes in L-PRP prepared by centrifugation. Colloids Surfaces B: Biointerfaces 161, 288–295. doi: 10.1016/j.colsurfb.2017.10.046, PMID: 29096373

[B10] Deppermann C. Kubes P. (2018). Start a fire, kill the bug: The role of platelets in inflammation and infection. Innate Immun. 24, 335–348. doi: 10.1177/1753425918789255, PMID: 30049243 PMC6830908

[B11] Drago L. Bortolin M. Vassena C. Romanò C. L. Taschieri S. Fabbro M. D. (2014). Plasma components and platelet activation are essential for the antimicrobial properties of autologous platelet-rich plasma: an *in vitro* study. PloS One 9, e107813. doi: 10.1371/journal.pone.0107813, PMID: 25232963 PMC4169456

[B12] Drago L. Bortolin M. Vassena C. Taschieri S. Del Fabbro M. (2013). Antimicrobial activity of pure platelet-rich plasma against microorganisms isolated from oral cavity. BMC Microbiol. 13, 47. doi: 10.1186/1471-2180-13-47, PMID: 23442413 PMC3599521

[B13] Edwards R. Harding K. G. (2004). Bacteria and wound healing. Curr. Opin. Infect. Dis. 17, 91–96. doi: 10.1097/00001432-200404000-00004, PMID: 15021046

[B14] Etulain J. (2018). Platelets in wound healing and regenerative medicine. Platelets 29, 556–568. doi: 10.1080/09537104.2018.1430357, PMID: 29442539

[B15] Fabbro M. D. Bortolin M. Taschieri S. Ceci C. Weinstein R. L. (2016). Antimicrobial properties of platelet-rich preparations. A systematic review of the current pre-clinical evidence. Platelets 27, 276–285. doi: 10.3109/09537104.2015.1116686, PMID: 26763769

[B16] Foster T. E. Puskas B. L. Mandelbaum B. R. Gerhardt M. B. Rodeo S. A. (2009). Platelet-rich plasma: from basic science to clinical applications. Am. J. Sports Med. 37, 2259–2272. doi: 10.1177/0363546509349921, PMID: 19875361

[B17] Gilbertie J. M. Schaer T. P. Schubert A. G. Jacob M. E. Menegatti S. Ashton Lavoie R. . (2020). Platelet-rich plasma lysate displays antibiofilm properties and restores antimicrobial activity against synovial fluid biofilms. vitro J. Orthop. Res. 38, 1365–1374. doi: 10.1002/jor.24584, PMID: 31922274 PMC8018705

[B18] Jenne C. N. Urrutia R. Kubes P. (2013). Platelets: bridging hemostasis, inflammation, and immunity. Int. J. Lab. Hematol. 35, 254–261. doi: 10.1111/ijlh.12084, PMID: 23590652

[B19] Li H. Hamza T. Tidwell J. E. Clovis N. Li B. (2013). Unique antimicrobial effects of platelet-rich plasma and its efficacy as a prophylaxis to prevent implant-associated spinal infection. Adv. Healthcare Materials 2, 1277–1284. doi: 10.1002/adhm.201200465, PMID: 23447088 PMC3774283

[B20] Liu W. Liu Y. Li T. Liu L. Du M. Du J. . (2024). Long-term stability of frozen platelet-rich plasma under –80 °C storage condition. Regenerative Ther. 26, 826–830. doi: 10.1016/j.reth.2024.09.006, PMID: 39329099 PMC11424891

[B21] Malone M. Schultz G. (2022). Challenges in the diagnosis and management of wound infection. Br. J. Dermatol. 187, 159–166. doi: 10.1111/bjd.21612, PMID: 35587707

[B22] Oudelaar B. W. Peerbooms J. C. Huis in ‘t Veld R. Vochteloo A. J. H. (2019). Concentrations of blood components in commercial platelet-rich plasma separation systems: A review of the literature. Am. J. Sports Med. 47, 479–487. doi: 10.1177/0363546517746112, PMID: 29337592

[B23] Patel A. N. Selzman C. H. Kumpati G. S. McKellar S. H. Bull D. A. (2016). Evaluation of autologous platelet rich plasma for cardiac surgery: outcome analysis of 2000 patients. J. Cardiothorac Surg. 11, 62. doi: 10.1186/s13019-016-0452-9, PMID: 27068030 PMC4828785

[B24] Percival S. L. Emanuel C. Cutting K. F. Williams D. W. (2012). Microbiology of the skin and the role of biofilms in infection. Int. Wound J. 9, 14–32. doi: 10.1111/j.1742-481X.2011.00836.x, PMID: 21973162 PMC7950481

[B25] Perego R. Meroni G. Martino P. A. Spada E. Baggiani L. Proverbio D. (2024). Antibacterial effect of canine leucocyte platelet-rich plasma (L-PRP) and canine platelet-poor plasma (PPP) against methicillin-sensitive and methicillin-resistant staphylococcus pseudintermedius. Veterinary Sci. 11, 670. doi: 10.3390/vetsci11120670, PMID: 39729010 PMC11680258

[B26] Scali C. Kunimoto B. (2013). An update on chronic wounds and the role of biofilms. J. Cutan Med. Surg. 17, 371–376. doi: 10.2310/7750.2013.12129, PMID: 24138971

[B27] Serraino G. F. Dominijanni A. Jiritano F. Rossi M. Cuda A. Caroleo S. . (2015). Platelet-rich plasma inside the sternotomy wound reduces the incidence of sternal wound infections. Int. Wound J. 12, 260–264. doi: 10.1111/iwj.12087, PMID: 23692143 PMC7950781

[B28] Sethi D. Martin K. E. Shrotriya S. Brown B. L. (2021). Systematic literature review evaluating evidence and mechanisms of action for platelet-rich plasma as an antibacterial agent. J. Cardiothorac Surg. 16, 277. doi: 10.1186/s13019-021-01652-2, PMID: 34583720 PMC8480088

[B29] Smith C. W. (2022). Release of α-granule contents during platelet activation. Platelets 33, 491–502. doi: 10.1080/09537104.2021.1913576, PMID: 34569425

[B30] Swanson T. Ousey K. Haesler E. Bjarnsholt T. Carville K. Idensohn P. . (2022). IWII Wound Infection in Clinical Practice consensus document: 2022 update. J. Wound Care 31, S10–S21. doi: 10.12968/jowc.2022.31.Sup12.S10, PMID: 36475844

[B31] Tohidnezhad M. Varoga D. Wruck C. J. Podschun R. Sachweh B. H. Bornemann J. . (2012). Platelets display potent antimicrobial activity and release human beta-defensin 2. Platelets 23, 217–223. doi: 10.3109/09537104.2011.610908, PMID: 21913811

[B32] Varshney S. Dwivedi A. Pandey V. (2019). Antimicrobial effects of various platelet rich concentrates-vibes from *in-vitro* studies-a systematic review. J. Oral. Biol. Craniofacial Res. 9, 299–305. doi: 10.1016/j.jobcr.2019.06.013, PMID: 31316893 PMC6611965

